# Supplementary material for: Vacuum Brazing of Metallized YSZ and Crofer Alloy Using 72Ag-28Cu Filler Foil
Source: Materials (Basel). 2022 Jan 26;15(3):939. doi: 10.3390/ma15030939 (PMC8838688; doi:10.3390/ma15030939)
Supplement: Supplementary file 1 [file materials-15-00939-s001.zip › materials-1527353-supplementary.pdf]

materials

Supplementary Material

Vacuum Brazing of Metallized YSZ and Crofer Alloy Using 72Ag-28Cu Filler Foil

Liang-Wei Huang [1,2], Ren-Kae Shiue [1,*], Chien-Kuo Liu [2], Yung-Neng Cheng [2], Ruey-Yi Lee [2] and Leu-Wen Tsay [3]

1 Department of Materials Science and Engineering, National Taiwan University, Taipei 10617, Taiwan; i13501350@iner.gov.tw
2 Nuclear Fuels and Materials Division, Institute of Nuclear Energy Research, Taoyuan 32546, Taiwan; ckliu2@iner.gov.tw (C.-K.L.); yncheng@iner.gov.tw (Y.-N.C.); rylee@iner.gov.tw (R.-Y.L.)
3 Department of Optoelectronics and Materials Technology, National Taiwan Ocean University, Keelung 20224, Taiwan; b0186@mail.ntou.edu.tw
* Correspondence: rkshiue@ntu.edu.tw

S1. The calibration of coated Cu layer thickness

The thicknesses of Cu coating layers were measured using the Alpha-Step D-500 stylus profiler (Figure S1). The thickness measurements for every sputtering power were conducted by 5 times in order to obtain the average thickness and its standard deviation. The correlation curve between average thickness and sputtering power was established by linear regression as shown in Figure S2. Consequently, the coated Cu thickness could be estimated in advance based on the correlation curve. For example, if the sputtering powers were 57 W and 172 W, the thicknesses of coated copper layers were 1 and 3 μm, respectively. The method was also applied to coated Ag and Ti layers in the experiment.

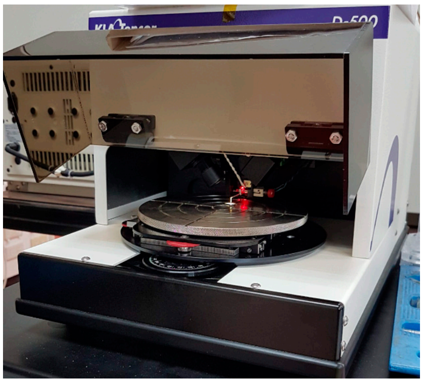

Figure S1. Alpha-Step D-500 stylus profiler (KLA, USA).

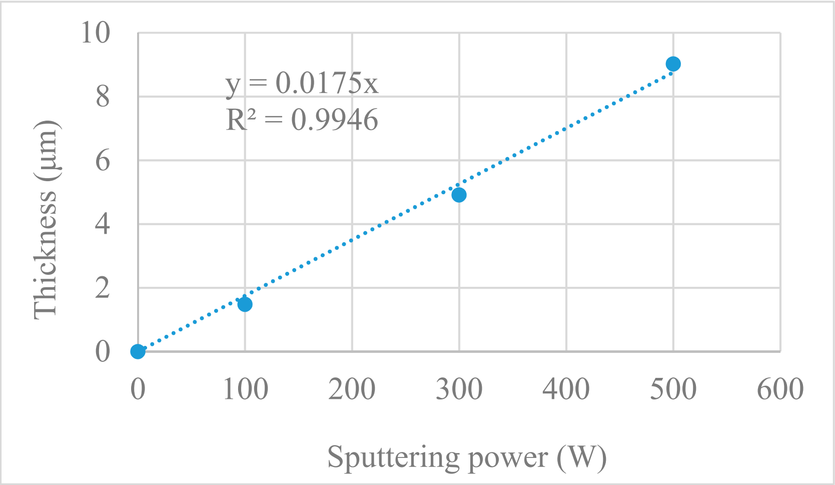

Figure S2. The correlation curve between Cu coating thickness and sputtering power.

S2. Assembly of the sample and the diagram of pressure drop test

The diagram of brazed sample and apparatus for the pressure drop test were illustrated in Figure S3 and Figure S4, respectively.

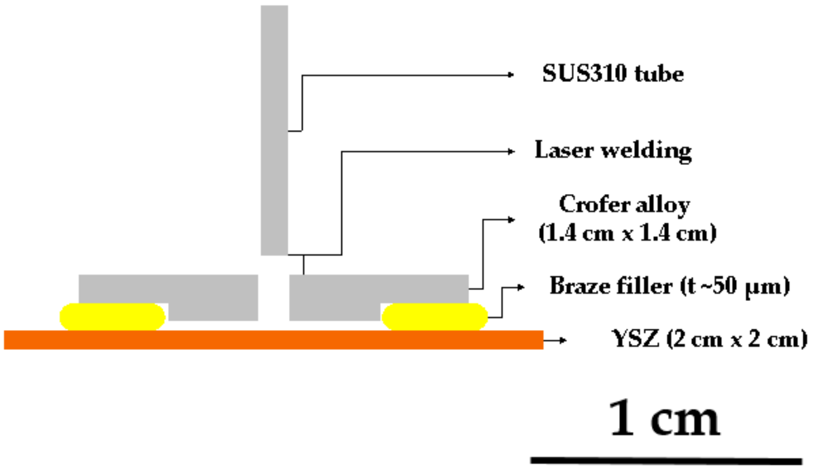

Figure S3. The assembly of brazed sample for the pressure drop test.

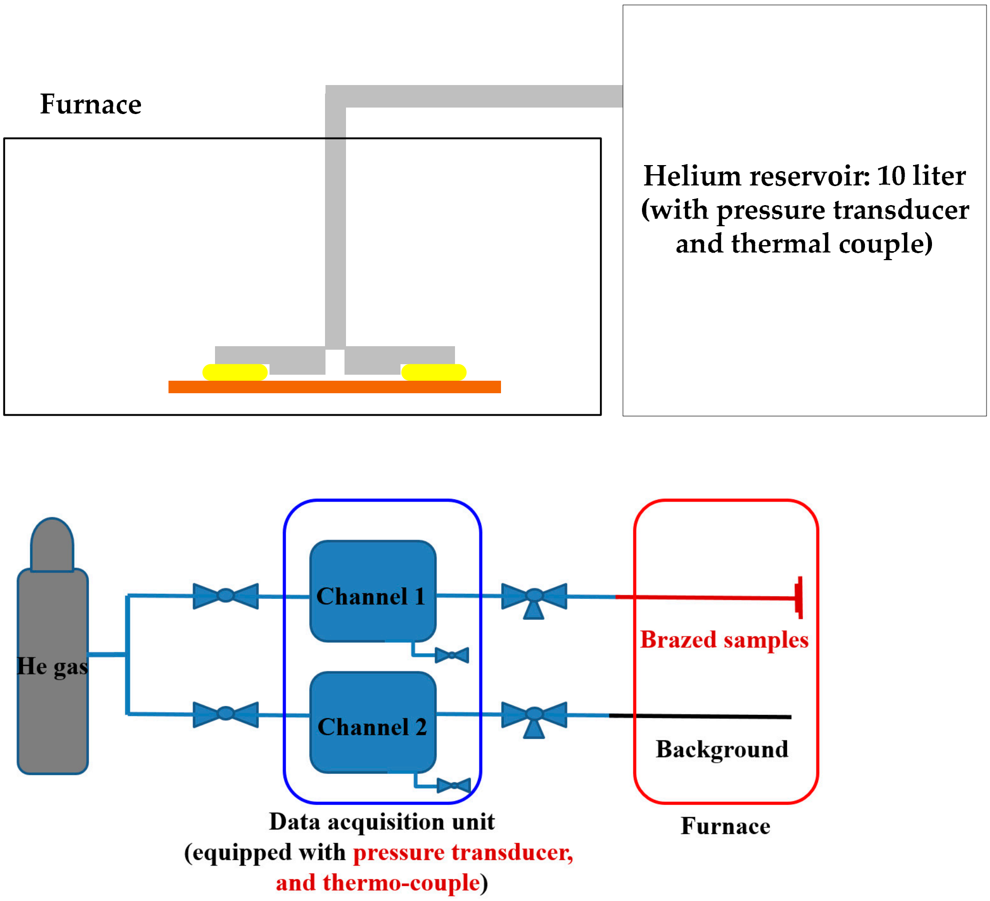

Figure S4. The apparatus of pressure drop test.
